# Molecular Signature of *Pseudomonas aeruginosa* with Simultaneous Nanomolar Detection of Quorum Sensing Signaling Molecules at a Boron-Doped Diamond Electrode

**DOI:** 10.1038/srep30001

**Published:** 2016-07-18

**Authors:** Alyah Buzid, Fengjun Shang, F. Jerry Reen, Eoin Ó Muimhneacháin, Sarah L. Clarke, Lin Zhou, John H. T. Luong, Fergal O’Gara, Gerard P. McGlacken, Jeremy D. Glennon

**Affiliations:** 1 Innovative Chromatography Group, Irish Separation Science Cluster (ISSC), Ireland; 2 Department of Chemistry and Analytical & Biological Chemistry Research Facility (ABCRF), University College Cork, Ireland; 3 BIOMERIT Research Centre, Department of Microbiology, University College Cork, Ireland

## Abstract

Electroanalysis was performed using a boron-doped diamond (BDD) electrode for the simultaneous detection of 2-heptyl-3-hydroxy-4-quinolone (PQS), 2-heptyl-4-hydroxyquinoline (HHQ) and pyocyanin (PYO). PQS and its precursor HHQ are two important signal molecules produced by *Pseudomonas aeruginosa*, while PYO is a redox active toxin involved in virulence and pathogenesis. This Gram-negative and opportunistic human pathogen is associated with a hospital-acquired infection particularly in patients with compromised immunity and is the primary cause of morbidity and mortality in cystic fibrosis (CF) patients. Early detection is crucial in the clinical management of this pathogen, with established infections entering a biofilm lifestyle that is refractory to conventional antibiotic therapies. Herein, a detection procedure was optimized and proven for the simultaneous detection of PYO, HHQ and PQS in standard mixtures, biological samples, and *P. aeruginosa* spiked CF sputum samples with remarkable sensitivity, down to nanomolar levels. Differential pulse voltammetry (DPV) scans were also applicable for monitoring the production of PYO, HHQ and PQS in *P. aeruginosa* PA14 over 8 h of cultivation. The simultaneous detection of these three compounds represents a molecular signature specific to this pathogen.

Bacterial species rely on a wide array of signaling molecules, signal detection systems, and signal-transduction mechanisms to coordinate gene regulation^1^. Quorum sensing (QS) is a cell-to-cell communication system, which involves the production and detection of diffusible signaling molecules, known as ‘autoinducers’ or bacterial pheromones^1^,^2^. Chemically diverse QS signaling molecules ranging from N-acyl homoserine lactones (AHLs) and 2-alkyl-4-quinolones (AHQs) to peptides and furanones, coordinate activities including bacterial secondary metabolite production, biofilm development, bioluminescence, competence, plasmid transfer and pathogenicity^2^,^3^. *Pseudomonas aeruginosa*, a Gram-negative opportunistic pathogen, is capable of surviving in a broad range of natural environments. It is an antibiotic-resistant human pathogen associated with hospital-acquired infections^4^ and causes acute pneumonia and chronic lung infections in cystic fibrosis (CF) patients^5^. Two AHL-based systems (the *las* and *rhl*) together with the AHQ-based system form a complex QS hierarchy in *P. aeruginosa* which controls global gene expression^6^. The primary *P. aeruginosa* AHQ signaling molecules are 2-heptyl-3-hydroxy-4-quinolone **1** (“Pseudomonas Quinolone Signal”, PQS) and its immediate precursor, 2-heptyl-4-hydroxyquinoline **2** (HHQ)^7^. Pyocyanin **3** (1-hydroxy-N-methylphenazine, PYO), one of the several phenazine-based secretory products of *P. aeruginosa* (Fig. 1), is also involved in QS^8^. PYO is also considered as an important virulence and pro-inflammatory factor. As a redox-active molecule, PYO is a potential source of damaging reactive species, which plays a significant role in oxidative stress and inducing lung injury infected by *P. aeruginosa*. In general, *P. aeruginosa* is the only species known to produce PQS and PYO while HHQ can be generated by *Burkholderia* strains^9^. Also, the lack of these molecules does not mean that *P. aeruginosa* is absent, since certain isolates lack a functioning AHQ system (e.g. *P. aeruginosa* PA7)^10^.

Among different analytical methods for AHQs and PYO detection, an LC-MS/MS method was developed to profile a broad range of QS signaling molecules including PQS and HHQ^3^. The PYO levels of patient sputa ranging from 0 to 27.3 μg mL^−1^ were detected by HPLC- UV detection^11^. Chromatography can be paired with mass spectrometry to enhance detection sensitivity, however, this approach requires sample pretreatment, lengthy analysis times and high costs, whereas fluorometry is sample-consuming and lacks selectivity^12^. Accurate and rapid point-of-care diagnosis has stimulated efforts to develop simple and sensitive electrochemical strategies. Biosensor-based assays for PQS and HHQ have been reported^2^,^13^,^14^ in addition to simple cyclic voltammetry (CV) and amperometry using a boron-doped diamond (BDD) thin-film electrode as an excellent method for the detection of HHQ, PQS^15^ and 2-(2-hydroxyphenyl)-thiazole-4-carbaldehyde (IQS)^16^. Adsorptive stripping voltammetry (AdSV) using a hanging mercury drop electrode (HMDE)^17^ and differential pulse voltammetry (DPV) using graphite rods^18^ were employed for PYO detection in biological samples. Disposable screen-printed electrodes were also used to probe the presence of PYO in the human biofluids by square wave voltammetry (SWV)^19^,^20^,^21^. Sismaet *et al*. measured the production of PYO in the presence of various amino acids. The presence of the amino acids resulted in a faster and stronger electrochemical response^22^. Kim *et al*.^23^ developed a bio-based redox capacitor for *in situ* monitoring of the production of PYO during *P. aeruginosa* cultivation. The catechol-grafted chitosan film amplified the electrochemical signals of PYO and lowered the detection limit. Miniaturized PYO sensors in the form of a ‘smart-bandage’^24^,^25^ or ‘nanofluidic’^8^,^26^ were also reported. There are, however, only two reports on the simultaneous electrochemical determination of PQS and PYO^27^,^28^. In the former, a conductive polymer film modified glassy carbon electrode has been applied to increase electroactive electrode surface. In the latter approach, a *P. aeruginosa* strain was grown on the electrode surface to concentrate electrochemical signals of PYO and PQS.

The BDD thin-film electrode^29^,^30^ featuring a wide potential range, high current density, extreme electrochemical stability, low background current and high resistance to fouling has proven promising for PQS detection^15^. This report unravels the use of a BDD electrode, without modification, for simultaneous determination of PYO, PQS and HHQ in a mixed solution. The influence of electrolyte pH on the peak potential separation and peak currents was also investigated. The optimized procedures were then applied to analyze supernatant extracts from *P. aeruginosa* wild-type strains, monitor the production of signaling molecules in the bacterial strain PA14 and detect the signals in CF patient sputa cultured with *P. aeruginosa* using a bare BDD electrode, taking advantages of the hydrogen surface termination and sp^3^ carbon bonding without the extended π-electron system for sensitive detection over other solid electrodes.

## Results and Discussion

### Electrochemical behavior of PQS, HHQ, and PYO

**Figure 2:**
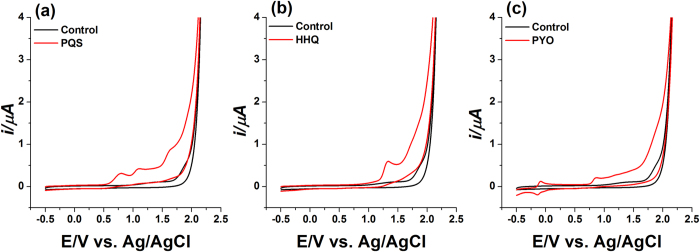
CV of (**a**) 50 μM PQS; (**b**) 50 μM HHQ; and (**c**) 10 μM PYO at 100 mV s^−1^ in 50 mM acetate buffer at pH 5.0 containing 20% ACN on the BDD electrode vs. Ag/AgCl. The black lines represent blank 50 mM acetate buffer at pH 5.0 containing 20% ACN.

The redox reactions of PYO and the quinolones on the BDD electrode were studied by CV. The CV obtained for PQS at pH 5.0 on the pristine BDD electrode exhibited three oxidation peaks at +0.759 V, +1.103 V and +1.590 V (Fig. 2a). In contrast, only one well-defined peak at +1.351 V was observed on the CV of HHQ (Fig. 2b), indicating the simultaneous voltammetric measurements of the quinolones is feasible due to the peak potential differentiation. The CV of PYO (Fig. 2c) presented a pair of redox peaks (oxidation at −0.1 V and reduction at −0.153 V) at the negative potential range, making these potentials unique for the identification/determination of PYO. PYO also provoked an oxidation peak at +0.871 V, resulting in a peak separation (∆Ep) of 112 mV to the first oxidation peak of PQS (+0.759 V). DPV was chosen for further experiments as a sensitive technique with good distinction against the background current.

### Effect of electrolyte pH on the detection of PYO, HHQ, and PQS

**Figure 3:**
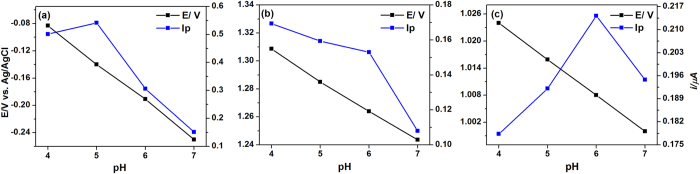
The effect of pH on the peak potential and peak current. DPV response the effect of pH (4.0 to 7.0) on the peak potential (Ep) and peak current (Ip) towards (**a**) 5 μM PYO; (**b**) 20 μM HHQ; and (**c**) 20 μM PQS on the BDD electrode vs. Ag/AgCl.

The influence of electrolyte pH on the peak potentials (Ep) and peak currents (Ip) of the electrooxidation of PYO, HHQ and PQS was investigated by DPV in the pH range of 4.0 to 7.0. As illustrated in Fig. 3a, the anodic peak potential for the oxidation of PYO shifted to negative values when increasing the buffer pH with linear regression of Ep (V) = 0.137–0.0551 (pH), indicating the role of protons in the oxidation process^31^. The determined slope was 55.1 mV/pH, close to the reported value of 61 mV/pH^27^ and the expected value of 59 mV/pH. Such behavior confirmed the number of hydrogen ions was equal to electrons taking part in the electrode reaction. The peak current (Ip) was also dependent on pH, which increased when the electrolyte pH decreased from 7.0 to 4.0. As protonation was involved in the catalytic reactions, the electrocatalytic oxidation of PYO became more favorable at lower pH^32^ thus resulting in higher peak current. However, the oxidation peak of PYO at pH 4.0 was 3.16% broader compared to the sharp peaks obtained at pH 5.0. The peak current intensity was decreased by increasing the pH to 6.0 and 7.0. The effect of buffer pH on the response of HHQ (Fig. 3b) and PQS (Fig. 3c) was investigated over the pH range 4.0 to 7.0. As a result of increasing pH, the peak potential shifted to less positive values.

**Figure 4:**
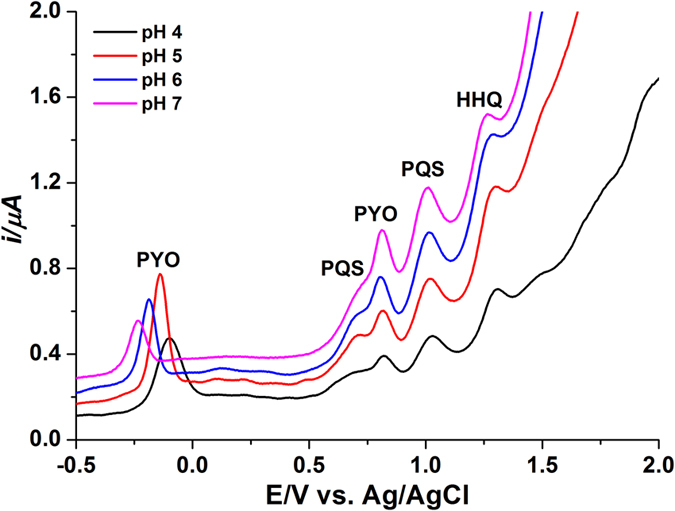
DPV of the ternary mixture of 5 μM PYO, 20 μM PQS and 20 μM HHQ at pH 4.0 to 7.0 on the BDD electrode vs. Ag/AgCl.

Figure 4 shows the DPVs for a ternary solution mixture of PYO, PQS and HHQ obtained with varying buffer pH. All the oxidation peaks of PQS and HHQ shifted to less positive values in the potential range of +0.5 V to +1.5 V and the oxidation peak of PYO shifted to more negative values in the potential range of −0.8 V to −0.25 V when increasing the pH from 4.0 to 7.0. The DPVs obtained at pH 5.0 and 6.0 exhibited five well-defined anodic peaks, whereas partially overlapped peaks of the biomarkers were observed in the voltammograms obtained at pH 4.0 and 7.0. The peak separations of PYO and PQS in the potential range of +0.65 V to +1.15 V were 138.9 mV, 176.2 mV, 170.5 mV and 161.1 mV for pH 4.0, 5.0, 6.0 and 7.0, respectively. The peak positions of the analytes might be attributed to different electrochemical activities of their functional groups on the electrode surface^33^. Similar to PYO, the oxidation peak currents of PQS and HHQ decreased with elevated pH. Therefore, considering the peak potential resolution and detection sensitivity, pH 5.0 acetate buffer (50 mM) was chosen as the optimal medium.

### Individual determination of PYO, PQS and HHQ

**Figure 5:**
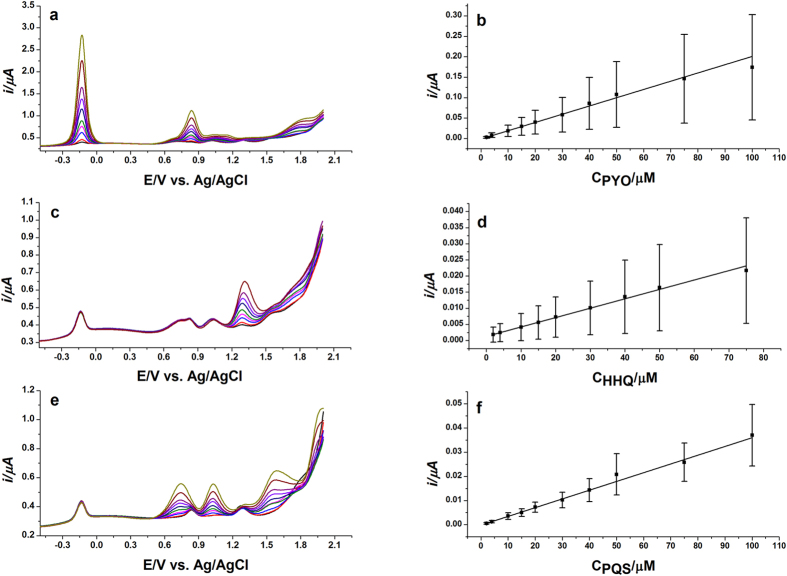
Calibration curve of PYO, HHQ and PQS. (**a**) DPVs of various PYO concentrations (1–100 μM) containing 10 μM HHQ and 20 μM PQS; (**b**) Calibration plot obtained from DPVs recording shown in (**a**); (**c**) DPVs of various HHQ concentrations (2–75 μM) containing 5 μM PYO and 20 μM PQS; (**d**) Calibration plot obtained from DPVs recording shown in (**c**); (**e**) DPVs of various PQS concentrations (1–100 μM) at BDD containing 5 μM PYO and 10 μM HHQ; (**f**) Calibration plot obtained from DPVs recording shown in **(e)**; Using the BDD electrode vs. Ag/AgCl in 50 mM acetate buffer, pH 5.0 containing 20% ACN. Error bars correspond to standard deviation (SD) (n = 3).

Considering sufficient peak potential difference and superb detection sensitivity in individual determinations, a series of studies was then performed to verify the feasibility of selective detection of PQS, HHQ and PYO by DPV using the BDD electrode. Experiments were carried out by changing the concentration of the target analyte whilst maintaining those of the other two species constant. Figure 5a shows the DPV response of PYO at different concentrations (2–100 μM) while PQS and HHQ were constant at 20 μM and 10 μM, respectively. The peak potential of PYO at −0.14 V remained unchanged when varying the concentrations of the compound. The current intensities of the oxidation peaks raised proportionally to increasing PYO concentration, in the range of 2–100 μM with good linearity (R^2^ = 0.991, Fig. 5b). The peak currents of PQS and HHQ decreased slightly without significant loss of electrocatalytic activities, due to the irreversible adsorption of the oxidation products on the electrode surface which hindered the further oxidation of PQS and HHQ on the electrode surface^34^. Similarly, Fig. 5c displays the DPV calibration of HHQ in the presence of PYO and PQS exhibiting satisfactory linearity in the range of 2–75 μM (R^2^ = 0.997, Fig. 5d). Also, Fig. 5e depicts the DPV calibration of PQS in the presence of PYO and HHQ demonstrating acceptable linearity in the range of 2–100 μM (R^2^ = 0.996, Fig. 5f). The oxidation peak of PQS ~+1.0 V was selected for calibration. The analytical parameters for simultaneous determination of PYO, HHQ and PQS are present in Table 1. The analytical performance of the BDD electrode using DPV is compared with the literature methods in Table 2. The effect of the pH and potential to obtain a lower limit of detection of PYO, HHQ and PQS are also investigated using amperometric measurement (*I/t*) and compared to DPV on the BDD electrode in Table 3. In the case of PQS and HHQ, there are two parameters that can result in lower LOD; the pH and the oxidation potential, for example, pH 2.0 gives sensitive LOD at +0.8 V and +1.2 V for PQS and HHQ, respectively. While in the PYO case, the oxidation potential at +1.0 V can result in lower LOD at either pH.

### Analysis of *P. aeruginosa* PA14 cultures, growth curves and clinical sample analysis

**Figure 6:**
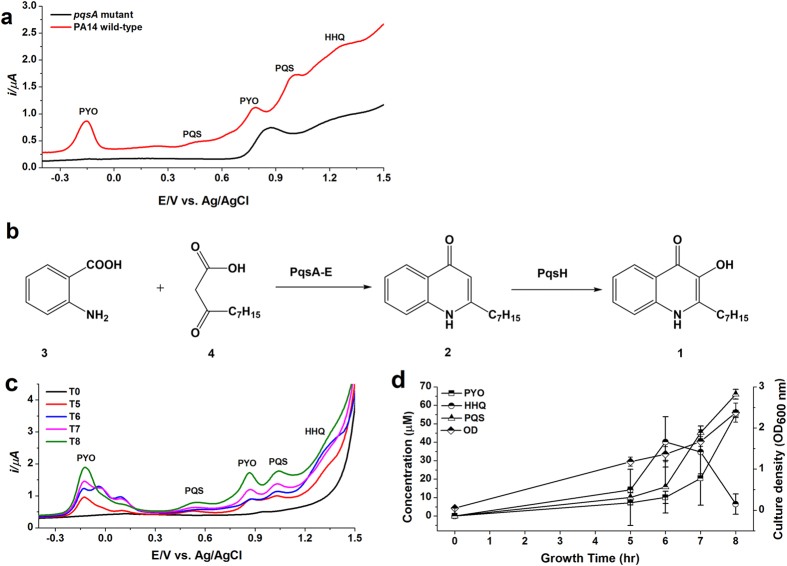
Bacterial cell-free culture PA14 analysis, HHQ and PQS biosynthesis routine and HHQ, PQS and PYO production kinetics in PA14. (**a**) DPVs of *P. aeruginosa* PA14 wild-type and *pqsA* mutant in LB media grown for 7 h at 37 °C with OD_600 nm_ of 2.4 and 2.1 for the PA14 wild-type and *pqsA* mutant, respectively. The cell-free culture was extracted twice with acidified ethyl acetate and a given amount of the reconstituted sample added to the electrolyte: 50 mM acetate buffer, pH 5.0 with 20% ACN. Detection on the BDD electrode vs. Ag/AgCl; (**b**) PQS biosynthesis in *P. aeruginosa*. Anthranilic acid 3 condenses with a β-keto fatty acid 4 to produce HHQ through the action of the *pqsABCDE* operon. The monooxygenase PqsH converts HHQ into PQS; (**c**) and (**d**) Monitoring the production of HHQ, PQS and PYO in the bacterial strain *P. aeruginosa* PA14 in LB media was carried out over 8 h. Cell density was measured in terms of OD_600 nm_. The bacterial culture was extracted twice with acidified ethyl acetate and MCX SPE, and a given amount of the eluent added to the electrolyte: 50 mM acetate buffer, pH 5.0 with 20% ACN. All measurements were made in triplicate, using peak area of the analytes response on the BDD electrode vs. Ag/AgCl. Error bars represent SD (n = 3). The oxidation peaks of −0.14 V and ~+1.0 V were presented for PYO and PQS, respectively.

The optimized method on DPV was initially applied to the analysis of cell-free culture (supernatant) extracts of *P. aeruginosa* PA14 and *pqsA* mutant. DPV of the biological sample extracts were recorded by diluting the samples into 50 mM acetate buffer (pH 5.0) containing 20% ACN. As expected, no analytes were detected from the supernatant of *P. aeruginosa pqsA* mutant. Nevertheless, *P. aeruginosa* PA14 produced a significant amount of PYO, in addition to the presence of PQS and HHQ (Fig. 6a). The concentration of PYO, HHQ and PQS measured in the cell-free extract of microbial strain PA14 were 37.03 ± 0.76 μM, 4.48 ± 0.43 μM, and 11.17 ± 0.15 μM, respectively (n = 3).

Subsequently, a time-course analysis was performed using the BDD electrode to monitor the real-time concentration profiles of the three target molecules from early log phase into the stationary phase of growth. Cultures were sampled at 1 h intervals from the mid-log phase and monitored as before. Consistent with the established kinetics of signal production in *P. aeruginosa*, HHQ was initially identified at the highest concentration^35^,^36^, not surprising given that it is the precursor to PQS (Fig. 6b)^37^. As the cells entered stationary phase, both PQS and PYO^38^ become more abundant, with HHQ levels markedly reduced at 8 h (Fig. 6c,d).

**Figure 7:**
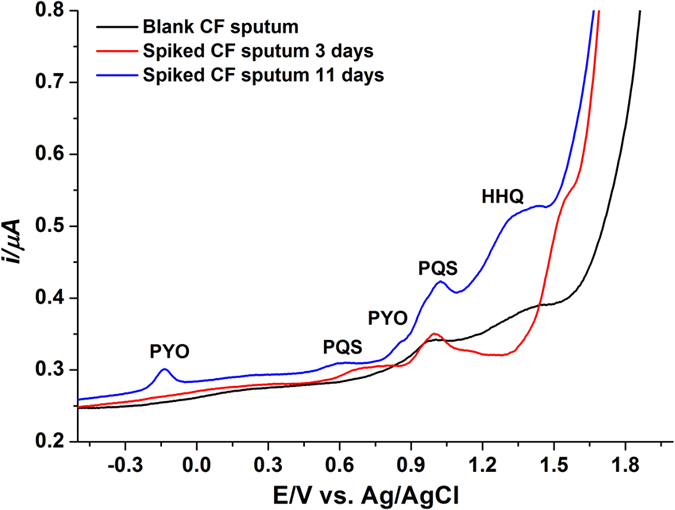
Clinical sample analysis. DPV of a CF sputum sample cultured with *P. aeruginosa* and incubated at 37 °C for 3 and 11 d. A small amount of sputum extract was added to the electrochemical cell containing 50 mM acetate buffer, pH 5.0 with 20% ACN and detected on the BDD electrode vs. Ag/AgCl.

CF sputum is a complex mixture of airway mucus glycoproteins, serum, proteins, DNA, alginate, and rigidifying lipids as well as inflammatory substances including polymorphonuclear leukocytes, antibodies, antimicrobial peptides, and dead host cells. Also, CF sputum samples are more viscous than normal samples as a result of negatively charged biopolymers (mucin, DNA, and alginate) which are connected through noncovalent interactions such as electrostatic, hydrogen, and hydrophobic bonding. All these matrix constituents protect the epithelial cells and form a diffusion barrier for pathogen and harmful particles^39^,^40^. In order to evaluate the extent of matrix interference, a sputum sample obtained from a paediatric CF patient who was not infected with *P. aeruginosa* was spiked with known amounts of PYO (10, 20, and 40 μM), HHQ (20, 40, and 80 μM), and PQS (40, 80, and 160 μM). All blank and spiked sputum samples were then extracted twice with chloroform (1:2, v/v sputum sample: chloroform). Importantly, the blank sputum sample did not produce any signature signal, consistent with the absence of *P. aeruginosa* signaling molecules. Therefore, the corresponding DPV exhibited no oxidation peaks. In the case of spiked sputum samples, all the target analytes were detected and recovery values measured were up to 32%, 43%, and 58% for PYO, HHQ, and PQS, respectively. The LOD of PYO, HHQ, and PQS in CF sputum sample using DPV on the bare BDD electrode is 0.15 μM, 0.62 μM, and 1.25 μM, respectively (S/N = 3). In addition, the sputum sample was spiked with 1 × 10^5^ cells of *P. aeruginosa* and incubated at 37 °C for several days to promote bacterial growth. The sputum sample was then extracted with chloroform, dried with a rotary evaporator and re-dispersed with ACN. The DPVs were recorded by adding a certain amount of the reconstituted sample into 50 mM acetate buffer (pH 5.0) containing 20% ACN. Figure 7 compares the DPV profiles of the blank and *P. aeruginosa* spiked sputum samples incubated for 3 d and 11 d. No peak obtained at the negative potential on the DPV curve of the sputum sample that was incubated for 3 d indicates the absence or a non-detectable amount of PYO. However, a clear peak at −0.14 V was exhibited on the DPV curve of the sample incubated for 11 d. A concentration of 2.8 μM, 7.2 μM, and 10.4 μM was calculated for PYO, HHQ, and PQS, respectively in spiked CF sputum sample incubated for 11 d. The target analytes were not detected in the sputum sample incubated for 3 d, likely due to a lower concentration of HHQ and PQS at this stage^41^ and the matrix effect.

## Conclusions

The BDD electrode was successfully used for simultaneous determination of PYO, HHQ and PQS in both standard mixtures and microbial *P. aeruginosa* strain PA14 using a conventional and rapid extraction method. The production of three target signals in *P. aeruginosa* PA14 over 8 h was monitored, illustrating the selectivity of BDD electrode with the obtained results in agreement with previous reports^35^,^36^,^38^. Most importantly, the application of the optimized method was extended to the CF sputum sample cultured with *P. aeruginosa*. In this regard, the importance of including the three biomarkers is highlighted by the recent suggestion that HHQ predominates over PQS in CF sputum samples^42^.

## Methods

### Chemicals and materials

All the reagents were of the analytical grade with the highest purity, and aqueous solutions were prepared in deionized water with a resistivity of 18.2 MΩ.cm (Millipore, Ireland). Pyocyanin, acetic acid, sodium acetate anhydrous, sodium phosphate dibasic, sodium phosphate monobasic, acetonitrile, methanol, 2-propanol, phosphoric acid, formic acid, ammonium formate, chloroform, and ethyl acetate were purchased from Sigma-Aldrich (Dublin, Ireland). Micro polish alumina powder (0.05 μm and 0.3 μm), nylon and masterTex polishing cloths were purchased from Buehler (Coventry, UK). Acetate buffer (50 mM, pH 4.0–6.0, with 20% ACN) and phosphate buffer (50 mM, pH 7.0, with 20% ACN) were employed as the supporting electrolytes. The stock solutions of 2 mM PQS, HHQ and PYO were prepared in ACN freshly before use. ACN was used to support solubility of the three target analytes. The solid-phase extraction was performed using the Oasis mixed-mode cation exchange cartridge MCX SPE 1 cc (30 mg) from Waters (Dublin, Ireland).

### Apparatus

Cyclic voltammetry (CV), differential pulse voltammetry (DPV) and amperometric measurement (I/t) were performed using a CHI1040A electrochemical workstation (CH Instrument, Austin, TX). The electrochemical cell consists of a BDD electrode as a working electrode (3 mm diameter, 0.1% doped boron, Windsor Scientific, Slough Berkshire, UK), an Ag/AgCl (3 M KCl) as a reference electrode (BASi Analytical Instruments, West Layette, IN) and a Pt wire as a counter electrode (Sigma-Aldrich, Dublin, Ireland). The convective transport during the amperometric determination was performed with magnetic stirring at 800 rpm. All pH values of different electrolytes were measured using a pH meter (a pH 210 microprocessor) which calibrated daily with standard buffer solutions. All measurements were performed at room temperature.

### Synthesis of PQS and HHQ

PQS and HHQ were synthesized as described by Pesci *et al*.^43^ and McGlacken *et al*.^44^. The resulting products and their purity were confirmed by ^1^H NMR (300 MHz) and spectra are shown in a previous publication^45^.

### Electrode preparation

The BDD electrode was polished with polishing papers (Buehler, UK) and subsequently with alumina (Buehler, UK) until a mirror finish was obtained. After cleaning the electrode with deionized water, the electrode was sonicated in 2-propanol and deionized water for 5 min and 10 min, respectively. Subsequently, the electrode was cleaned by CV between −1.0 V and +2.0 V versus Ag/AgCl (3 M KCl) at 100 mV s^−1^ in 50 mM acetate buffer (pH 5.0) until a stable CV profile was obtained.

### Bacterial strains, growth conditions and clinical samples

Bacterial supernatant extracts were obtained using a modified version of the Fletcher protocol^2^. Briefly, overnight cultures of *P. aeruginosa* PA14 were transferred into fresh Luria-Bertani (LB) broth (OD_600nm_ 0.01) and incubated for 7 h at 37 °C (total 20 mL). Culture supernatants were obtained by centrifugation at 4000 revolutions per minute (rpm) for 15 min and subsequently filter sterilized using Minisart (Sartorius) 0.2 μm filter into a clean centrifuge tube. Extractions using acidified ethyl acetate (0.01% (v/v) glacial acetic acid) were performed twice on bacterial cell-free cultures (end-point assays) (1:2, v/v supernatants: acidified ethyl acetate) or whole cell cultures (time-point assays) (1:1, v/v whole cell culture: acidified ethyl acetate). In both assays, the organic phase was evaporated using a rotary evaporator and the residue was dissolved in ACN (end-point assay) and in 0.5 M formic acid pH 2.0 (time-point assay). Solid-phase extraction using MCX SPE was carried out after liquid-liquid extraction for time-point assays. The reconstituted samples were subjected to MCX SPE cartridges pre-conditioned with methanol and deionized water. The cartridges were washed then with 0.5 M formate buffer, pH 2.0 to increase the retention of analytes and washed with 100% methanol to remove neutral interferences. Subsequently, the analytes were eluted using 5% 4.5 M ammonium formate in methanol.

### Ethics statement and sputum processing

Sputum samples were collected from paediatric patients attending the CF clinic at Cork University Hospital, Ireland. Ethical approval was granted by the Clinical Research Ethics Committee (CREC) for sputum collection and samples were handled in accordance with the approved guidelines. All methods were carried out in accordance with the approved guidelines. Written informed consent from all patients/guardians was obtained for acquisition and analysis outlined in this study. Patient sputum samples were inoculated with 1 × 10^5^ cells of *P. aeruginosa* and incubated at 37 °C for 3 and 11 d, respectively. CF patient samples were extracted twice with chloroform (1:2, v/v sputum sample: chloroform). As mentioned above, the organic phase was evaporated using a rotary evaporator and the residue was dissolved in ACN.

## Additional Information

**How to cite this article**: Buzid, A. *et al*. Molecular Signature of *Pseudomonas aeruginosa* with Simultaneous Nanomolar Detection of Quorum Sensing Signaling Molecules at a Boron-Doped Diamond Electrode. *Sci. Rep.* **6**, 30001; doi: 10.1038/srep30001 (2016).

## Figures and Tables

**Figure 1:**
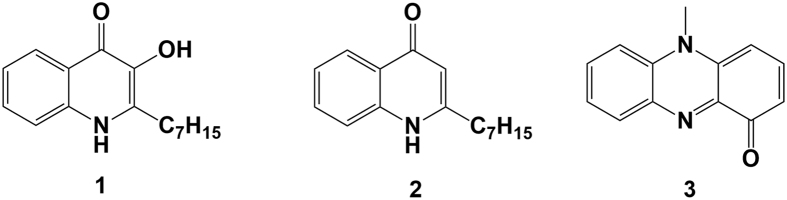
The structure of *P. aeruginosa* AHQ and PYO signals.

**Table 1:** Calibration curve and limit of detection using DPV at the BDD electrode.

Analytes	Linear range (μM)	Linear regression equation (I: μA, C: μM)	Correlation coefficient (R^2^)	R.S.D % (n = 3)^a^	Limit of detections (LODs)^b^
PYO	2–100	I _pyo_ = 2.019 × 10^−9^C − 1.190 × 10^−9^	0.991	1.73	50 nM
HHQ	2–75	I _HHQ_ = 2.913 × 10^−10^C + 1.278 × 10^−9^	0.996	2.45	250 nM
PQS	2–100	I _PQS_ = 3.615 × 10^−10^C − 1.78 × 10^−10^	0.995	1.18	250 nM

^a^R.S.D (%) calculated from triplicated DPV measurements for the potential at 20 μM each of PYO, HHQ, and PQS (n = 3).

^b^LOD (*S/N* = *3*).

**Table 2:** Comparison of the proposed method on the BDD electrode using DPV with the literature methods for simultaneous detection of PYO, HHQ, and PQS.

Method	Analyte	Linear range (μM)	Correlation coefficient (R^2^)	Detection limit (nM)	Reference
BDD	PYO	2–100	0.991	50 nM	Present work
	HHQ	2–75	0.996	250 nM	
	PQS	2–100	0.995	250 nM	
BDD	PQS	0.1–5.5	0.999	1 nM	15
HMDE	PYO	0.002–0.3	0.99	2 nM	17
Catechol-chitosan/Gold electrode	PYO	0.1–1	N.R^a^	50 nM	22
Carbon fibre tow	PYO	1–100	0.998	30 nM	24
NEAs/Gold electrode^b^	PYO	0–100	0.96	441 nM	26
NEAs/Gold electrode	PYO	1–100	0.941	597 nM	8

^a^N.R, not reported.

^b^NEAs, nanofluidic electrode assemblies.

**Table 3:** Comparison of the limit of detection of the three signals between DPV and *I/t* curve on the BDD electrode.

Analyte	0.8 V, *I/t*,pH 2.0	1.0 V, *I/t*,pH 2.0	1.2 V, *I/t*,pH 2.0	0.8 V, *I/t*,pH 5.0	1.0 V, *I/t*,pH 5.0	1.2 V, *I/t*,pH 5.0	DPV, pH 5.0
PYO	600 nM	40 nM	—	400 nM	40 nM	—	50 nM
HHQ	200 nM	—	16 nM	32000 nM	—	160 nM	250 nM
PQS	1 nM	—	—	32 nM	—	—	250 nM
